# Utility of quantitative pathologic analysis of pT1 colorectal carcinomas to improve prediction of lymph node metastasis

**DOI:** 10.1007/s00428-025-04284-2

**Published:** 2025-10-08

**Authors:** Priya Nayak, Heidi Kosiorek, Reetesh K. Pai, Sameer Shivji, Catherine E. Hagen, Rondell P. Graham, Daniel D. Buchanan, Mark A. Jenkins, Amanda I. Phipps, Loic Le Marchand, Christina Wu, Niloy J. Samadder, Carol J. Swallow, Steven J. Gallinger, Robert C. Grant, Thomas Westerling-Bui, James Conner, David P. Cyr, Richard Kirsch, Rish K. Pai

**Affiliations:** 1 Department of Pathology and Laboratory Medicine, Mayo Clinic Arizona, Scottsdale, AZ USA; 2 Department of Quantitative Health Sciences, Mayo Clinic, Phoenix, AZ USA; 3 Department of Pathology, University of Pittsburgh Medical Center, Pittsburgh, PA USA; 4 Department of Pathology and Laboratory Medicine, Mount Sinai Hospital, 600 University Ave, Toronto, ON Canada; 5 Department of Pathology and Laboratory Medicine, Mayo Clinic, Rochester, MN USA; 6 Colorectal Oncogenomics Group, Department of Clinical Pathology, The University of Melbourne, Parkville, VIC Australia; 7 University of Melbourne Centre for Cancer Research, Victorian Comprehensive Cancer Centre, Parkville, VIC Australia; 8 Genomic Medicine and Family Cancer Clinic, Royal Melbourne Hospital, Parkville, VIC Australia; 9 Melbourne School of Population and Global Health, Centre for Epidemiology and Biostatistics, The University of Melbourne, Carlton, VIC Australia; 10 Public Health Sciences Division, Fred Hutchinson Cancer Center, Seattle, WA USA; 11 Department of Epidemiology, University of Washington, Seattle, WA USA; 12 Population Sciences in the Pacific Program (Epidemiology), University of Hawaii, Honolulu, HI USA; 13 Division of Medical Oncology, Department of Medicine, Mayo Clinic, Phoenix, AZ USA; 14 Division of Gastroenterology and Hepatology, Department of Medicine, Mayo Clinic, Phoenix, AZ USA; 15 Sinai Health System, Lunenfeld-Tanenbaum Research Institute, Toronto, ON Canada; 16 Department of Surgical Oncology, Princess Margaret Cancer Centre, Mount Sinai Hospital, Toronto, ON Canada; 17 Division of General Surgery, Department of Surgery, University of Toronto, Toronto, ON Canada; 18 Institute of Medical Science, University of Toronto, Toronto, ON Canada; 19 Ontario Institute for Cancer Research, Toronto, ON Canada; 20 Surgical Oncology Program, University Health Network, Toronto, ON Canada; 21 Division of Medical Oncology and Hematology, Princess Margaret Cancer Centre, Toronto, ON Canada; 22 Aiforia Inc., Cambridge, MA USA; 23 Department of Laboratory Medicine and Pathobiology, University of Toronto, Toronto, ON Canada

**Keywords:** Quantitative digital pathology, Colorectal cancer, Lymph node metastasis, Malignant polyp

## Abstract

According to the National Comprehensive Cancer Network (NCCN), submucosally invasive (pT1) colorectal carcinomas (CRCs) should be evaluated for tumor grade, lymphatic invasion, and tumor budding to determine the risk of lymph node metastasis. The presence of any one of these high-risk features is an indication for surgery in endoscopically removed pT1 CRCs. In this study, we determined if quantitative pathologic analysis with the QuantCRC algorithm can augment NCCN risk stratification in a multi-institutional cohort of 512 surgically resected pT1 CRC. LASSO regression identified %high-grade, %inflammatory stroma (stromal area), and %tumor budding/poorly differentiated clusters (%TB/PDC) as important QuantCRC features and were used in subsequent logistic regression analysis. Five logistic regression models were built using NCCN and QuantCRC variables, with the combined NCCN + QuantCRC model providing the highest Area Under the Curve (AUC) of 0.74 (95% CI 0.68–0.81). A predicted probability cutoff of 0.092 provided a sensitivity of 78.3% and specificity of 62.1% in the NCCN + QuantCRC model with a 24.3% rate of lymph node positivity for high-risk (HR) tumors compared to 5.2% for low-risk (LR) CRCs. Fifteen pT1 CRCs were reclassified from NCCN LR to NCCN + QuantCRC HR and 3/15 (20%) demonstrated lymph node positivity. The median predicted probability of lymph node metastasis in the NCCN + QuantCRC model was used to define two HR groups (HR1: 0.092–0.218 and HR2: > 0.218). HR2 CRCs had a rate of lymph node positivity of 31.5% compared to 17.1% for HR1 CRCs (*P* = 0.02). Lastly, the NCCN + QuantCRC model was validated in a cohort of 29 endoscopically resected pT1 CRCs followed by surgical resection. In the NCCN + QuantCRC model, the 8 pN + CRCs in this cohort had a higher median predicted probability of lymph node metastasis compared to 21 pN0 CRCs (0.219 vs. 0.080, *P* = 0.04). In summary, the addition of variables from QuantCRC can improve risk stratification of pT1 CRCs over NCCN criteria alone.

**Supplementary information:**

The online version contains supplementary material available at 10.1007/s00428-025-04284-2.

## Introduction

The majorityof submucosally invasive colorectal carcinomas (CRCs) have the potential to be cured by local resection [1–6]. The National Comprehensive Cancer Network (NCCN) clinical practice guidelines [6] for both colon and rectal cancer recognize that patients with pT1 cancers within an endoscopically removed polyp can be observed if favorable pathologic features are present (version 6.2024 for colon cancer and version 5.2024 for rectal cancer; see https://www.nccn.org). Unfavorable, or high-risk, pathologic features include positive margin, poor differentiation (G3), and lymphatic invasion (LI). The NCCN also recognizes tumor budding as an additional high-risk feature that “may preclude polypectomy as an adequate treatment of endoscopically removed malignant polyps” [6].

Poor differentiation has been shown to increase the risk of lymph node metastasis and has a prevalence between 2 and 7% in pT1 tumors [7–12]. Tumor involvement of lymphatics is another well-known feature associated with an increased risk of lymph node metastasis [7–9, 11, 13, 14]. In a meta-analysis of 67 studies of pT1 CRCs, LI was associated with an odds ratio of 3.16 (95% CI 1.88–5.33) for positive lymph nodes [13]. Numerous studies have also shown the effects of tumor budding on the risk of lymph node metastasis with both grades Bd2 and Bd3 (as assessed by the International Tumor Budding Consensus Conference [15]) being associated with lymph node positivity [9, 10, 13, 16–18] and confirmed in a recent meta-analysis with an odds ratio of 2.83 (95% CI 2.06–3.88) [13]. In summary, there is considerable evidence to include tumor grade, LI, and tumor budding in risk stratification schemes despite the interobserver variability that exists among pathologists in evaluating these features [19–21]. For these reasons, LI, tumor grade, and tumor budding are also recognized as important for risk stratification by numerous organizations [1, 5, 22, 23].

Multiple other pathologic, endoscopic, immunohistochemical, and molecular features have been studied to improve risk stratification in pT1 tumors. Depth of submucosal invasion has been the most well-studied additional pathologic feature [8–10, 17, 24], with the most commonly used cutoff of > 1000 mm. However, a recent large meta-analysis demonstrated that deep submucosal invasion alone was associated with only a 2.6% prevalence of lymph node metastasis and was not a predictor of lymph node involvement in multivariate analysis [13]. Poorly differentiated clusters (PDCs) are defined as non-gland-forming clusters of five or more tumor cells and represent a similar process as tumor budding [25]. PDCs have been associated with lymph node metastasis in some studies [16, 26]. Invasive carcinomas in pedunculated polyps were thought to have a lower risk of lymph node metastasis compared to sessile polyps; however, tumor morphology was not associated with the risk of lymph node metastasis in one large study and endoscopic configuration is specifically not included in NCCN risk stratification [6, 9].

With advances in digital pathology, there is tremendous interest in harnessing data present in histologic images to predict outcomes. We recently developed and validated a quantitative segmentation algorithm, QuantCRC, to segment an image of CRC into 15 different regions and objects that correspond to known pathologic features [27, 28]. We have demonstrated that the quantitative output from this algorithm has significant associations with pathologist-derived features and molecular alterations [28, 28–30]. Moreover, the quantitative data were able to improve prediction of recurrence-free survival in multiple cohorts [29, 31]. In this study, we evaluated the ability of QuantCRC to improve prediction of lymph node metastasis.

## Materials and methods

### Cohort description

**Fig. 1:**
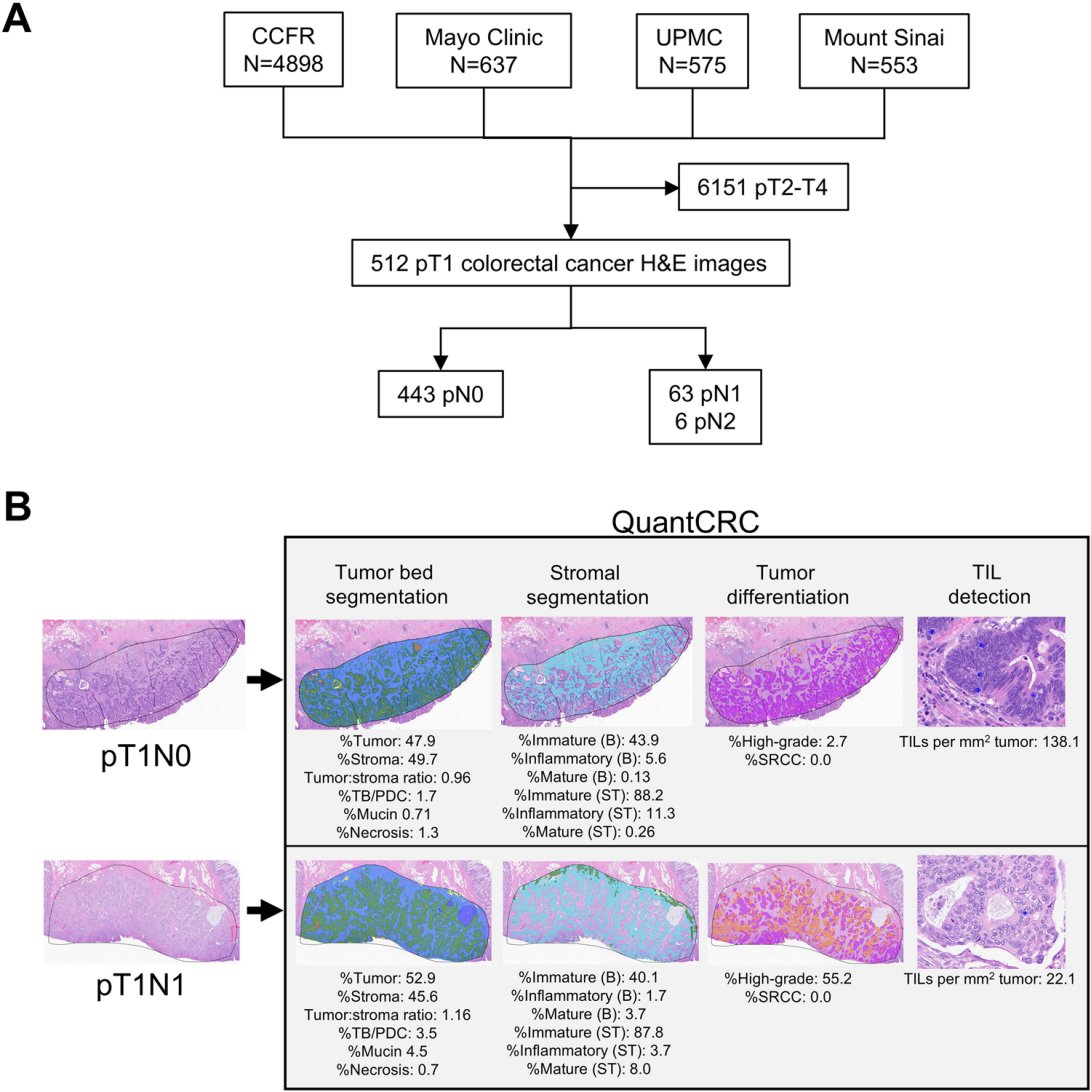
Cohort details and application of QuantCRC. **A** Flow chart depicting cohorts used in this study and number of CRCs digitized from the four cohorts. **B** QuantCRC was applied to the cohort of pT1 CRCs. QuantCRC segments the image in a stepwise manner. First the image is segmented into carcinoma (green), stroma (light blue), mucin (dark blue), TB/PDC (red), necrosis (brown), smooth muscle (purple), and fat (yellow). Next the stroma is segmented into immature (teal), mature (green), and inflammatory (gray). The carcinoma is segmented into low-grade (purple), high-grade (orange), and signet ring cell (light green). Finally, TILs are recognized as objects (blue dots) within the tumor epithelium. After this segmentation, fifteen features are calculated from each image. Two pT1 CRCs (one pN0 and one pN +) are shown

The study cohort consisted of non-neoadjuvant treated and surgically resected CRCs from the Colon Cancer Family Registry (*N* = 4898) and three additional sites: University of Pittsburgh Medical Center (UPMC) (*N* = 575), Mount Sinai Hospital (*N* = 553), and Mayo Clinic (*N* = 637) (Fig. 1A). The CCFR enrolled participants after CRC diagnosis with prospective follow-up and has been described in great detail [32, 33]. The UPMC and Mount Sinai consisted of consecutively resected CRCs at these institutions between 2010 and 2015 and 2011 and 2016, respectively. The Mayo Clinic cohort consisted of consecutively resected CRC from 2017 to 2024 (*N* = 637). From this cohort, a total of 512 pT1 CRCs were identified for downstream analysis, of which 443 were pN0, 63 were pN1, and 6 were pN2.

To evaluate the performance of the logistic regression models, a cohort of endoscopically resected polyps with pT1 CRCs that had subsequent colorectal resections at the Mayo Clinic and UPMC (*N* = 29) was collected, of which 8 were pN1 and 21 were pN0. The study was approved by the institutional review board (IRB 18–11309 and 22–001404).

### Pathologic review and National Comprehensive Cancer Network risk stratification

The following data were extracted: age, sex, tumor location, lymph node status, histologic grade, and presence or absence of tumor within lymphatics (lymphatic invasion). Re-review of the tumor was performed to obtain the grade of tumor budding (Bd1, Bd2, and Bd3). Depth of invasion was measured on the digitized image according to criteria by Ueno et al. [17], and 1000 mm was used as a cutoff to define deep submucosal invasion.

### Application of QuantCRC

An H&E-stained slide containing the tumor was digitized using Leica Aperio GT450 or AT2 at × 40 magnification. The images were uploaded to the Aiforia Create deep learning cloud-based platform (Aiforia Technologies, Helsinki, Finland). For each image, the tumor bed was outlined and QuantCRC was applied. QuantCRC was developed in 2021 [27] and further refined and validated in 2022 [28]. QuantCRC has been used in multiple subsequent studies to segment an image of CRC to provide quantitative data for downstream analyses [29–31]. The unmodified QuantCRC algorithm from our prior publications was used in this study and is available from Aiforia, Inc. for research use only. Details on algorithm development and validation are described in *Gastroenterology *2022 Dec;163(6):1531–1546 [28]. The following fifteen QuantCRC parameters were recorded for each tumor: %tumor, %stroma, tumor:stroma ratio, %TB/PDC within the tumor, %mucin within the tumor, %necrosis within the tumor bed, %high-grade, %SRCC, TILs per mm^2^ of tumor epithelium, %immature stroma (tumor bed), %inflammatory stroma (tumor bed), %mature stroma (tumor bed), %immature (stromal region), %inflammatory (stromal region), and %mature (stromal region).

### Statistical analysis and logistic regression modeling

Continuous variables were summarized using median (IQR), and categorical variables were summarized using the frequency. Chi-squared, Fischer’s exact, and Wilcoxon rank-sum tests were used to compare categorical data. Kruskal–Wallis test was used for categorical and continuous QuantCRC variables. Least absolute shrinkage and selection operator (LASSO) regression was used to select QuantCRC predictor variables of lymph node metastasis. Subsequently, logistic regression was performed, and five models (using backward selection) were evaluated: (1) NCCN risk stratification (LR vs. HR), (2) QuantCRC variables selected by LASSO regression, (3) individual NCCN features (grade, LI, and Bd), (4) individual NCCN features and LASSO-selected QuantCRC features, and (5) LASSO-selected QuantCRC features and NCCN risk stratification (LR vs. HR). A comparison of models was done using the Akaike’s Information criteria (AIC) criteria and bootstrap optimism-corrected AUC. The predicted probability corresponding to the maximum Youden statistic for each model provided the cut-off for optimal sensitivity and specificity. Predicted probabilities from the logistic regression model were examined to identify low- and high-risk groups. *P*-values < 0.05 were significant. R version 4.2.2, Python version 3.11.8, and SPSS version 28.0 were used for statistical analysis.

## Results

### Details of the study cohort

From 6,151 CRC H&E images, 512 surgically resected pT1 CRCs were identified (Fig. 1A and Table 1). There were significant differences in the distribution of G3 CRCs between pN0 (8.8%) and pN + (20.3%). LI (40.6%) and Bd2/3 (55.1%) were more frequent in pN + tumors (both *P* < 0.001). There was no difference in median depth of invasion. Moreover, no differences were seen when depth of invasion was stratified by < 1000 mm or > 1000 mm.

**Table 1:** Clinical and pathologic parameters of the study cohort

Clinical and pathologic parameters	All pT1 CRCs	pT1N0	pT1N +	*P*-value
Female sex, %	45.1	45.1	45.5	0.96
Median age (IQR)	62 (18)	63 (19)	59 (19)	0.16
Tumor location, *N* (%)				
Cecum	77 (15.4)	71 (92.2)	6 (7.8)	0.1
Ascending colon	103 (20.6)	95 (92.2)	8 (7.8)	
Transverse colon	48 (9.6)	41 (85.4)	7 (14.6)	
Descending colon	25 (5.0)	20 (80.0)	5 (20.0)	
Sigmoid colon	103 (20.6)	86 (83.5)	17 (16.5)	
Rectum	143 (28.7)	118 (82.5)	25 (17.5)	
Unknown	13	12	1	
Median lymph nodes evaluated (IQR)	16 (21)	16 (21)	18 (20)	0.8
MMR status, *N* (%)				
MMRP	388 (77.3)	329 (76.0)	59 (85.5)	0.079
MMRD	114 (22.7)	104 (24.0)	10 (14.5)	
MMR status unknown	10	10	0	
pN stage, *N* (%)				
pN0	443 (86.5)	443 (100)	0	N.A
pN1	63 (12.3)	0	63 (91.3)	
pN2	6 (1.2)	0	6 (8.7)	
Histologic grade, *N* (%)				
G1/2	459 (89.6)	404 (91.2)	55 (79.7)	0.004
G3	53 (10.4)	39 (8.8)	14 (20.3)	
Lymphatic invasion, *N* (%)				
Absent	424 (82.8)	383 (86.5)	41 (59.4)	< 0.001
Present	88 (17.2)	60 (13.5)	28 (40.6)	
Tumor budding grade, *N* (%)				
Bd1	360 (70.3)	329 (74.2)	31 (44.9)	< 0.001
Bd2	79 (15.4)	59 (13.3)	20 (29.0)	
Bd3	73 (14.3)	55 (12.4)	18 (26.1)	
Median depth of invasion (IQR) (mm)	3.0 (2.3)	2.9 (2.3)	3.3 (2.3)	0.068
Depth of invasion risk, *N* (%)				
Low risk (< 1000 mm)	71 (13.9)	64 (14.1)	7 (10.1)	0.34
High risk (> 1000 mm)	441 (86.1)	379 (85.6)	62 (89.9)	
Overall NCCN risk category, *N* (%)				
Low risk	305 (59.6)	287 (64.8)	18 (26.2)	< 0.001
High risk	207 (40.4)	156 (35.2)	51 (73.9)	

Abbreviations: *IQR*, interquartile range; *MMRD*, mismatch repair deficiency; *MMRP*, mismatch repair proficiency; *NCCN*, National Comprehensive Cancer Network

In this cohort, 207 (40.4%) pT1 CRCs were NCCN-high risk (HR) and 305 NCCN-low risk (LR). Of the 69 pN + tumors in this cohort, 51 (73.9%) were NCCN-HR. To evaluate the effect of having one or more than one NCCN-HR feature on pN status, tumors were categorized into having 1, 2, or 3 HR features (Supplemental Table 1). In this cohort, there was no difference in rates of lymph node metastasis among tumors with 1, 2, or 3 NCCN-HR features (*P* = 0.08). Furthermore, there was no difference in the proportion of pN + in CRCs that were characterized by G3 only (6/21, 28.6%), LI only (7/29, 24.1%), or Bd2/3 only (13/83, 15.7%) (*P* = 0.3). Similarly, there was no difference in the proportion of pN + among the 62 tumors with various combinations of 2 NCCN-HR features (*P* = 0.2).

### Association of QuantCRC with NCCN risk stratification and pN status

For each CRC, QuantCRC was applied to extract quantitative data for 15 features (Fig. 1B). There were statistically significant differences in 11/15 QuantCRC among NCCN risk groups (Table 2). When stratified by pN status, there were significant differences in four QuantCRC features including %TB/PDC, %high-grade, TILs per mm^2^, and %inflammatory stroma (stromal area) (Table 2).

**Table 2:** QuantCRC features according to NCCN risk classification and pN status

QuantCRC features, median	NCCN LR (*N* = 305)	NCCN HR (*N* = 207)	*P*-value	pN0 (*N* = 443)	pN + (*N* = 69)	*P*-value
%Tumor (IQR)	54.7 (17.7)	50.1 (17.2)	< 0.001	52.7 (17.3)	51.3 (17.4)	0.4
%Stroma (IQR)	40.9 (17.6)	45.9 (17.3)	< 0.001	42.1 (16.9)	44.2 (18.9)	0.5
%Mucin within tumor (IQR)	1.0 (8.5)	0.7 (2.7)	0.03	0.9 (5.3)	0.7 (4.6)	0.4
%Necrosis (IQR)	2.4 (4.0)	2.1 (3.1)	0.07	2.3 (3.7)	2.3 (4.0)	0.7
%TB/PDC (IQR)	0.5 (0.8)	1.4 (2.1)	< 0.001	0.7 (1.1)	1.2 (2.5)	< 0.001
Tumor: stroma ratio (IQR)	1.3 (1.1)	1.1 (0.8)	< 0.001	1.2 (1.0)	1.1 (1.0)	0.5
TILs per mm^2^ (IQR)	75.9 (104.2)	49.6 (73.7)	< 0.001	68.2 (99.9)	53.5 (64.1)	0.03
%High grade (IQR)	6.9 (13.9)	13.2 (20.1)	< 0.001	8.1 (14.9)	15.6 (20.7)	< 0.001
%SRCC (IQR)	0.03 (0.2)	0.02 (0.2)	0.7	0.02 (0.2)	0.02 (0.1)	0.8
%Immature stroma, tumor bed (IQR)	28.3 (16.6)	36.7 (15.9)	< 0.001	31.8 (17.1)	32.7 (15.6)	0.1
%Inflammatory stroma, tumor bed (IQR)	6.2 (9.1)	5.1 (8.0)	0.02	5.7 (9.2)	4.9 (7.4)	0.09
%Mature stroma, tumor bed (IQR)	1.0 (2.3)	1.2 (1.7)	0.5	1.1 (2.0)	1.1 (2.3)	0.9
%Immature stroma, stromal area (IQR)	76.6 (24.5)	83.7 (19.9)	< 0.001	79.8 (23.2)	82.6 (20.9)	0.1
%Inflammatory stroma, stromal area (IQR)	16.3 (21.4)	11.1 (17.2)	< 0.001	14.2 (20.9)	12.2 (17.6)	0.04
%Mature stroma, stromal area (IQR)	2.7 (5.5)	2.6 (3.9)	0.5	2.7 (4.8)	3.0 (5.6)	1.0

Abbreviations: *HR*, high risk; *IQR*, interquartile range; *NCCN*, National Comprehensive Cancer Network; LR, low risk; *TB/PDC*, tumor budding/poorly differentiated clusters; *TIL*, tumor infiltrating lymphocyte

### Logistic regression models using QuantCRC and NCCN variables

LASSO regression was used to select the QuantCRC variables to include in subsequent logistic regression models. %High-grade, %TB/PDC, and %inflammatory stroma (stromal area) had non-zero LASSO coefficients of 0.0167, 0.0655, and − 0.0173 respectively, consistent with their observed association with lymph node status.

We compared five logistic regression models: NCCN risk category, LASSO-selected QuantCRC features, individual NCCN features, individual NCCN features + LASSO-selected QuantCRC features, and NCCN risk category + LASSO-selected QuantCRC features. The output of each model was the predicted probability of pN + for each CRC, and the AUC for each model is shown in Table 3. During backwards selection of the model including all NCCN risk features and QuantCRC, NCCN grade and NCCN Bd score were dropped from the model, leaving only pathologist-derived LI and QuantCRC %high-grade, %TB/PDC, and %inflammatory stroma (LI + QuantCRC), indicating that QuantCRC measures of grade and tumor budding are better predictors of lymph node metastasis than pathologist-derived measurements of these features. We also measured the performance of each model using the Akaike information criterion (AIC), with lower values indicating better performance. The NCCN + QuantCRC model provided the highest AUC and lowest AIC (Table 3).

**Table 3:** Comparison of logistic regression models using NCCN and QuantCRC variables

Model	pN +		Specificity	Sensitivity	AUC (95% CI)	AIC
	LR	HR				
QuantCRC	23/309 (7.4%)	46/203 (22.7%)	64.6%	66.7%	0.68 (0.61–0.75)	388.9
NCCN risk category	18/305 (5.9%)	51/207 (24.6%)	64.8%	73.9%	0.69 (0.64–0.75)	371.9
Grade, Bd, LI	18/305 (5.9%)	51/207 (24.6%)	64.8%	73.9%	0.71 (0.65–0.78)	378.0
LI + QuantCRC	20/321 (6.2%)	49/191 (25.7%)	68.0%	71.0%	0.72 (0.66–0.80)	371.3
NCCN + QuantCRC	15/290 (5.2%)	54/222 (24.3%)	62.1%	78.3%	0.74 (0.68–0.81)	368.4

*Abbreviations: AUC*, area under the curve; *AIC*, Akaike information criterion; *Bd*, tumor budding; *HR*, high risk; *LI*, lymphatic invasion; *LR*, low risk; *NCCN*, National Comprehensive Cancer Network

Given that the NCCN + QuantCRC model provided the best overall performance, this model was explored in more detail. The range and relative frequencies of predicted probabilities for the 512 pT1 CRCs in the NCCN + QuantCRC model are shown in Supplemental Fig. 1. A predicted probability cutoff of 0.092 in the NCCN + QuantCRC model provided a sensitivity of 78.3% and specificity of 62.1%. All NCCN + QuantCRC-LR were categorized as NCCN-LR, and 15/290 (5.1%) were pN +. All NCCN-HR CRCs were categorized as NCCN + QuantCRC-HR, with 51/207 (24.6%) demonstrating lymph node metastasis. However, 15 NCCN-LR CRCs were reclassified as NCCN + QuantCRC-HR, of which 3/15 (20%) were pN +.

**Fig. 2:**
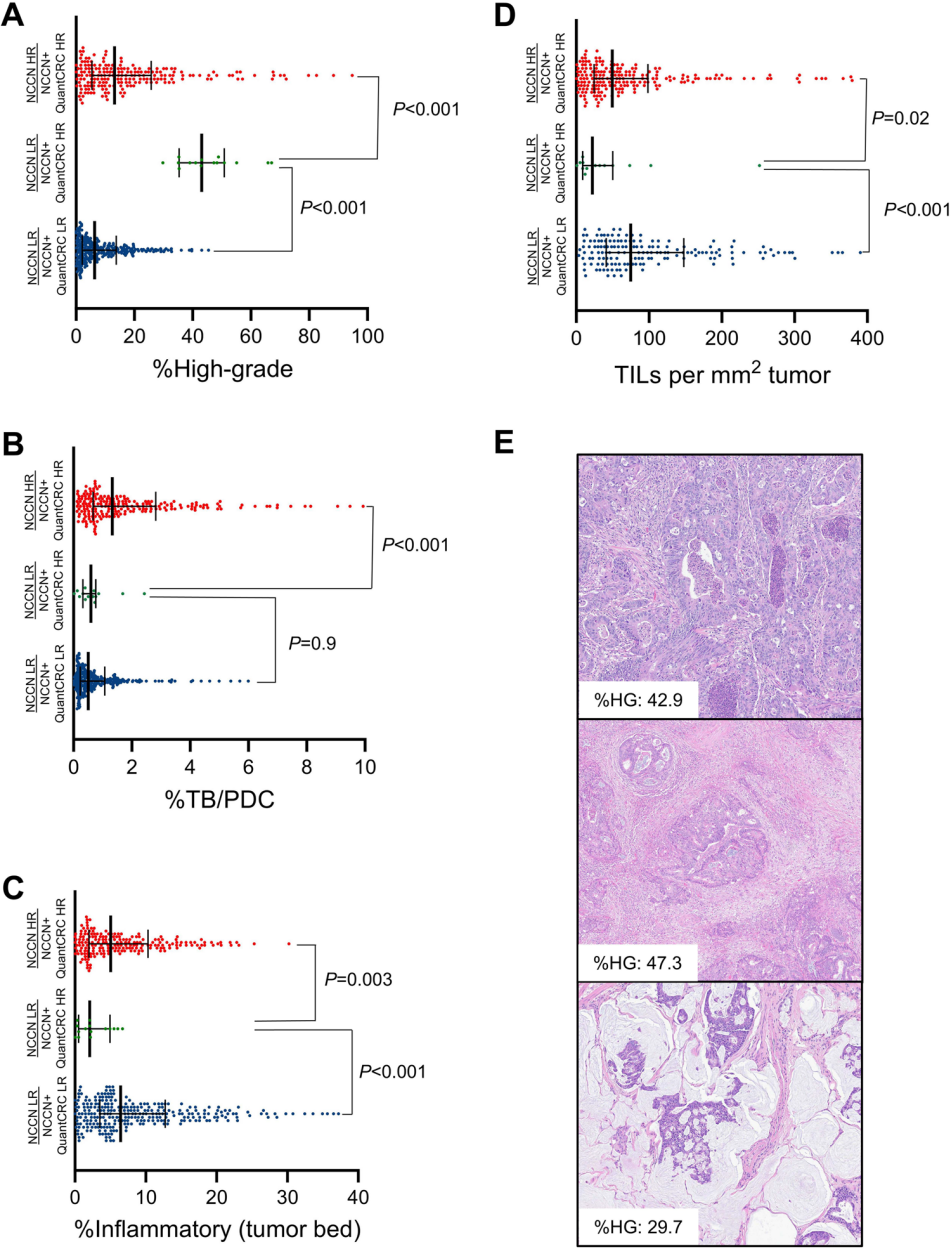
Comparison of the NCCN and NCCN + QuantCRC models. **A–D** Scatter plots of four QuantCRC features between NCCN LR/NCCN + QuantCRC LR, NCCN LR/NCCN + QuantCRC HR, and NCCN HR/NCCN + QuantCRC HR pT1 CRCs. **E** Hematoxylin and eosin images of the three pT1 CRCs with lymph node metastasis that were originally classified as NCCN LR but NCCN + QuantCRC HR

QuantCRC variables were compared between the NCCN-LR/NCCN + QuantCRC-LR (*N* = 290), NCCN-LR/NCCN + QuantCRC-HR (*N* = 15), and NCCN-HR/NCCN + QuantCRC-HR (*N* = 207) groups (Supplemental Table 2). Compared with the NCCN-LR/NCCN + QuantCRC-LR group, the NCCN-LR/NCCN + QuantCRC-HR group had lower TILs per mm^2^, higher %high-grade, lower %inflammatory stroma (tumor bed), higher %immature stroma (stromal area), and lower %inflammatory stroma (both tumor bed and stromal area). Compared with the NCCN-HR/NCCN + QuantCRC-HR group, the NCCN-LR/NCCN + QuantCRC-HR group had lower %TB/PDC, higher tumor:stroma ratio, lower TILs per mm^2^, higher %high-grade, and lower %inflammatory stroma (both tumor bed and stromal area). Figure 2A–D illustrates the differences in selected QuantCRC features among these groups. The histology of the 3 pN + cases in the 15 cases classified as NCCN-LR but NCCN + QuantCRC-HR was reviewed, and two demonstrated adenocarcinomas with cribriform histology (Fig. 2E). The other CRC was a mucinous adenocarcinoma with clusters of tumor cells floating in mucin pools with areas of cribriform architecture.

Given that the logistic regression models provide a predicted probability of lymph node metastases, we evaluated if this value could be further used to risk stratify pT1 CRCs to identify those at highest risk of having pN +. The median value of predicted probabilities in the NCCN + QuantCRC-HR group was used to define two HR groups: HR1 with predicted probabilities between 0.092 and 0.218 and HR2 with predicted probabilities > 0.218. Table 4 shows the differences between these two HR groups in regard to pN status and NCCN risk factors. The HR2 group identifies tumors at highest risk with 46/111 (31.5%) in this group demonstrating pN + compared to 19/111 (17.1%) in the HR1 group and 5.2% in the NCCN + QuantCRC-LR group. The tumors in the HR2 group were more likely to be G3 (30.6%) and had a higher frequency of Bd2/3 (80.1%). They were also more likely to have multiple NCCN high-risk factors compared with the HR1 group. Significant differences were seen in QuantCRC features between the NCCN + QuantCRC-HR1 and HR2 groups (Supplemental Table 3). In addition to the expected differences in %high-grade, %inflammatory stroma, and %TB/PDC given that these were included in the model, differences in %immature stroma and TILs per mm^2^ were also seen in HR1 vs. HR2 groups (Supplemental Fig. 2).

**Table 4:** Lymph node status and NCCN risk features in the NCCN + QuantCRC high-risk group stratified by predicted probability of lymph node metastasis

QuantCRC features	NCCN + QuantCRC HR1 [probability = 0.092–0.218] (*N* = 111), *N* (%)	NCCN + QuantCRC HR2 [probability > 0.218] (*N* = 111), ***N*** (%)	*P*-value
pN status			
pN0	92 (82.9)	76 (68.5)	0.02
pN +	19 (17.1)	46 (31.5)	
Histologic grade			
G1–2	92 (82.9)	77 (69.4)	0.03
G3	19 (17.1)	34 (30.6)	
Lymphatic invasion			
Absent	70 (63.1)	64 (57.7)	0.5
Present	41 (36.9)	47 (42.3)	
Tumor budding grade			
Bd1	48 (43.2)	22 (19.8)	< 0.001
Bd2	35 (31.5)	44 (39.6)	
Bd3	28 (25.2)	45 (40.5)	
NCCN risk factors			
None	15 (13.5)	0 (0)	< 0.001
1	71 (64.0)	62 (55.9)	
2	23 (20.7)	39 (35.1)	
3	12 (1.8)	10 (9.0)	

Abbreviations: *Bd*, tumor budding; *HR*, high risk; *NCCN*, National Comprehensive Cancer Network

### Logistic regression model performance on endoscopically resected pT1 CRCs

To validate the logistic regression models, 29 endoscopically resected pT1 CRCs were collected, of which 21 were pN0 and 8 were pN + after subsequent surgical resection. There were no differences in NCCN risk category (*P* = 0.09) between pN0 and pN + cases (Supplemental Table 4). Despite there being 16 endoscopic resections having positive or indeterminate margins, none had residual tumor at the site of endoscopic resection in the surgical specimen.

**Fig. 3:**
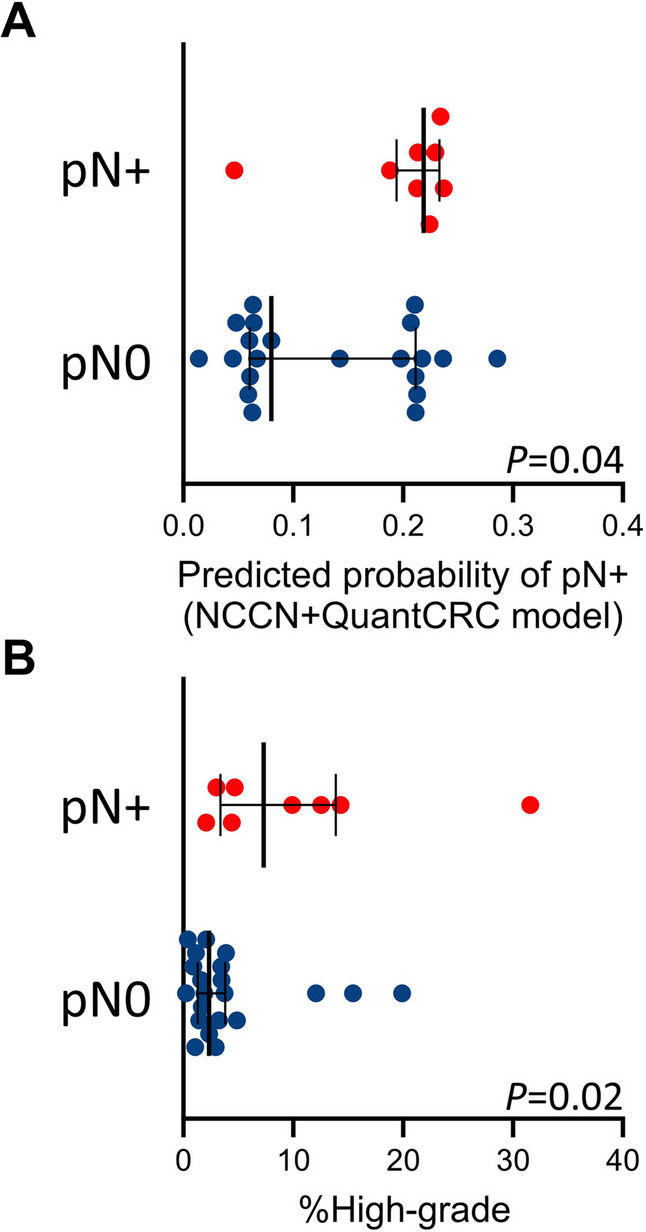
Model performance in the validation cohort of endoscopically resected pT1 CRC followed by subsequent surgical resection. **A** Scatter plot of the predicted probability of lymph node metastasis according to the NCCN + QuantCRC model in pN0 and pN + endoscopically resected pT1 CRCs. **B** QuantCRC %high-grade in the validation cohort according to lymph node status in endoscopically resected pT1 CRCs

There were no differences in the predicted probability of lymph node metastasis in the QuantCRC, Grade/Bd/LI, and LI + QuantCRC logistic regression models. There was a significant difference in the median predicted probability of lymph node metastasis in the NCCN + QuantCRC model with a predicted probability of 0.080 (IQR 0.150) for pN0 and 0.219 (IQR 0.039) for pN + CRCs in this validation cohort (*P* = 0.04) (Fig. 3A). Significant differences in QuantCRC %high-grade were observed between pN0 and pN + CRCs (Fig. 3B). According to the NCCN + QuantCRC model, 11 (52.4%), 8 (38.1%), and 2 (9.5%) pN0 CRCs were categorized as LR, HR1, and HR2 respectively. In contrast, 1 (12.5%), 3 (37.5%), and 4 (50%) pN + CRCs were categorized as LR, HR1, and HR2 respectively (*P* = 0.03).

## Discussion

Identifying submucosally invasive CRC with a high risk of lymph node metastasis is an area of intense research given the increasing use of advanced endoscopic and surgical techniques to remove large polyps in the colon and rectum [1, 2, 4, 14, 22, 34]. Numerous pathologic, immunohistochemical, and molecular features have been studied, with the NCCN risk stratification scheme being commonly used [6]. The NCCN risk stratification scheme relies on pathologic evaluation of LI, tumor budding, and tumor grade to determine the risk of lymph node metastasis and margin status to determine the risk of residual tumor at the site of endoscopic resection. These features are used to determine the need for additional surgery in endoscopically resected pT1 CRCs. One goal of this study was to determine if quantitative pathologic data extracted from a digitized image have associations with lymph node status. Additionally, we wanted to determine if the addition of quantitative pathologic data can augment current NCCN risk stratification for lymph node metastasis in pT1 CRCs. Given that our goal was predicting lymph node metastasis, we used a cohort of surgically resected pT1 CRCs with known pN status to evaluate five logistic regression models.

Four QuantCRC features had significant associations with lymph node status, including %TB/PDC, TILs per mm^2^, %high-grade, and %inflammatory stroma (stromal area). pT1 CRCs with lymph node metastasis had higher %TB/PDC and %high-grade, consistent with known associations between pathologist-derived evaluation of tumor grade, tumor budding, and lymph node status. The %TB/PDC also includes measurement of PDCs, which is an added benefit of QuantCRC, given that PDCs have been associated with pN + in some studies [16, 26]. In contrast, pN0 tumors had higher levels of TILs per mm^2^ and %inflammatory stroma (stromal area) compared to pN + tumors, consistent with known associations between a rich immune microenvironment and tumor behavior [18, 29, 35–37]. These associations confirm that quantitative analysis of pT1 CRC with QuantCRC identifies features associated with lymph node status. Furthermore, these features are biologically consistent with what is known about CRC behavior.

Given these findings, we evaluated the ability of five logistic regression models to predict lymph node status: QuantCRC, NCCN risk category, Grade/Bd/LI, LI + QuantCRC, and NCCN + QuantCRC. The NCCN + QuantCRC model provided the highest AUC and sensitivity for predicting lymph node positivity. Additionally, of the 15 CRCs that were originally classified as LR in the NCCN model but HR in the NCCN + QuantCRC model, three (20%) had lymph node metastasis, which is similar to the overall percentage of pN + in the NCCN-HR group (24.6%). These three patients would have been missed if NCCN criteria were used to risk stratify tumors. These three tumors had a median QuantCRC %high-grade of 43.1% compared to 6.9% for NCCN LR tumors despite being categorized as G1/2. Re-review of the histology of these three tumors identified cribriform architecture in all three tumors, which can be difficult to grade [20]. Cribriform histology has been identified in some studies as an adverse pathologic growth pattern, including in pT1 tumors [38, 39].

One important benefit of the NCCN + QuantCRC model is the ability to provide more granular information regarding the risk of pN + as the output is a predicted probability of lymph node positivity. Using the median value of predicted probabilities in the NCCN + QuantCRC-HR population, we demonstrate that this model can identify patients at very high risk of pN +. Of the 111 NCCN + QuantCRC-HR CRCs with predicted probability above the median of 0.218, 46/111 (31.5%) had lymph node metastasis compared to 19/111 (17.1%) of NCCN + QuantCRC-HR tumors with predicted probabilities between 0.092 and 0.218. The predicted probability of a given tumor can be weighed against the risk of surgery in a patient, particularly in those with multiple co-morbidities. This is in contrast with the NCCN criteria which provide only a binary estimate of risk. We validated this approach using a cohort of 29 endoscopically resected pT1 CRCs that underwent subsequent surgical resection. Only the NCCN + QuantCRC model was able to distinguish between pN0 and pN + CRCs in this group. Furthermore, 4/8 (50%) pN + CRCs in the validation cohort were classified as NCCN + QuantCRC-HR2 in contrast to only 1/21 (9.5%) pN0 CRC. Moreover, only 1/8 pN + tumors was classified as LR in contrast to 11/21 (52.4%) of the pN0 tumors.

Other studies have employed digital pathology to risk stratify early invasive CRC. Brockmoeller et al. developed a deep learning algorithm using 203pT1 tumors to predict lymph node metastasis, of which 16% had pN + [40]. The AUC for predicting any lymph node metastasis was 0.567, which is lower than all models developed in the present study. Another group developed a deep learning model to identify the peritumoral stroma in CRC and tested the ability of the peritumoral stroma score to predict lymph node metastasis in 164 CRCs from the Cancer Genome Atlas [41]. The model achieved an AUC of 0.677, but the study was not restricted to pT1 tumors. In contrast, our model that incorporates NCCN risk category and LASSO-selected QuantCRC features achieved an AUC of 0.74.

Other pT1 risk calculators have been developed, with one calculator assessing 6 pathologic features to arrive at an estimated risk, including tumor grade, Haggitt level, tumor budding, PDCs, LI, and the status of the muscularis mucosae to achieve an AUC of 0.83 [16]. While useful, PDC and the status of the muscularis mucosae are features not routinely reported by pathologists. Furthermore, the calculator was intended for pedunculated polyps only. A recent model was developed using depth of invasion, tumor grade, and LI as assessed by a group of 8 gastrointestinal pathologists and an automated measurement of tumor buds by immunohistochemistry, and it achieved an AUC of 0.64, lower than the NCCN + QuantCRC model [18]. Recently, a nomogram from Japan has been developed that includes tumor location, gender, depth of invasion, LI, and tumor budding, and it achieved a concordance index of 0.790 [42]. In this model, depth of invasion > 2000 um is considered the strongest feature associated with lymph node metastasis, which is not consistent with a recent large meta-analysis [13]. A more recent study from Australia provided a nomogram based on age, gender, polyp location, depth of invasion, lymphovascular space invasion, tumor grade, polyp type, mismatch repair immunohistochemistry, and margin status to determine the risk of lymph node metastasis or residual tumor, with an AUC of 0.76. While powerful, the specific risk of lymph node metastasis was not separated in this model, and direct comparison with our NCCN + QuantCRC model is not possible [43].

One strength of this study is the large number of pT1 tumors analyzed. Additionally, the CRCs came from four cohorts, which model generalizability. Another benefit of including the QuantCRC algorithm is that it was trained in a supervised manner to quantify interpretable features and has been used in multiple prior studies [27–29, 31, 44]. Thus, the features used in the NCCN + QuantCRC model have biologic relevance and are consistent with known pathologic features in CRC. Lastly, we were able to validate the NCCN + QuantCRC model in a cohort of endoscopically resected pT1 CRCs.

However, our study has some notable limitations and weaknesses. First, the combined NCCN + QuantCRC model demonstrated only modest improvements over the current NCCN scheme. While any improvement over current practice is helpful, the modest improvement coupled with the need to digitize the image while still requiring pathologist evaluation of NCCN features may limit adoption. Second, data on tumor grade and LI were abstracted from the pathology reports, which may make these data less reliable than if these features were assessed by a panel of expert gastrointestinal pathologists. Third, we did not specifically assess model performance on pT1 rectal cancers, which have unique therapeutic options including transanal excision. Fourth, our validation cohort was quite small and consisted of only 8 pN + CRCs. Furthermore, the validation cohort was selected to be enriched in pN + cases and is not fully representative of the target population to which this model would be applied. We are currently in the process of identifying a larger cohort of endoscopically resected pT1 CRCs with known lymph node status to apply this model.

In summary, we demonstrate that quantitative pathologic analysis of digitized images of pT1 CRC identifies features that are associated with lymph node status. Incorporation of three QuantCRC features improves the ability of the NCCN risk stratification scheme to identify high-risk pT1 CRCs. The model also provides a predicted probability of lymph node metastasis that has advantages when weighing the risk of surgery with the risk of observation alone.

## Supplementary information

Below are the links to the electronic supplementary materials.

Supplementary file 1 (PDF 701 KB)

Supplementary file 2 (DOCX 37.7 KB)

## Data Availability

The CCFR data, including the images created for this study, can be requested at www.coloncfr.org. The UPMC, Mayo Clinic, and Mount Sinai data are available from the authors on reasonable request.

## Abbreviations

CRC: Colorectal carcinoma
IQR: Interquartile range
NCCN: National Comprehensive Cancer Network
TB/PDC: Tumor budding/poorly differentiated clusters
SRCC: Signet ring cell carcinoma
TIL: Tumor infiltrating lymphocyte
