# Supplementary material for: Utility of quantitative pathologic analysis of pT1 colorectal carcinomas to improve prediction of lymph node metastasis
Source: Virchows Arch. 2025 Oct 8;488(6):1319–30. doi: 10.1007/s00428-025-04284-2 (PMC13264598; doi:10.1007/s00428-025-04284-2)
Supplement: Supplementary file 1 — (PDF 701 KB) [file 428_2025_4284_MOESM1_ESM.pdf]

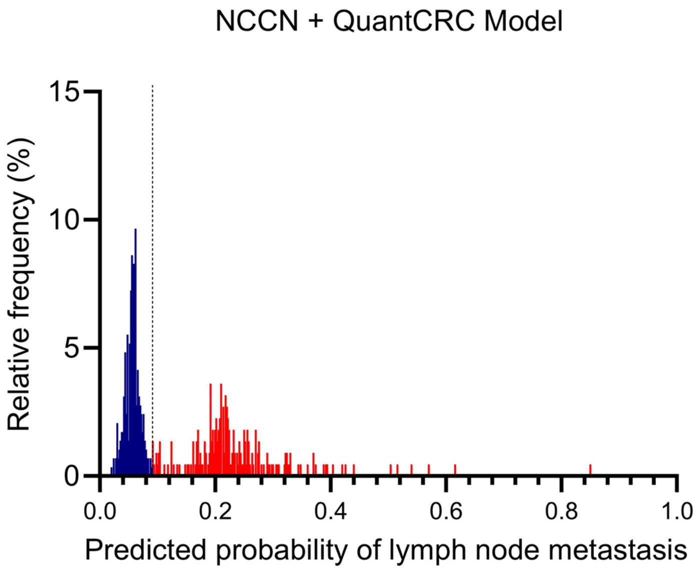

Supplemental Figure 1. Logistic regression model. Frequency distribution of the predicted probability of lymph node metastasis from the NCCN+QuantCRC model in the study cohort.

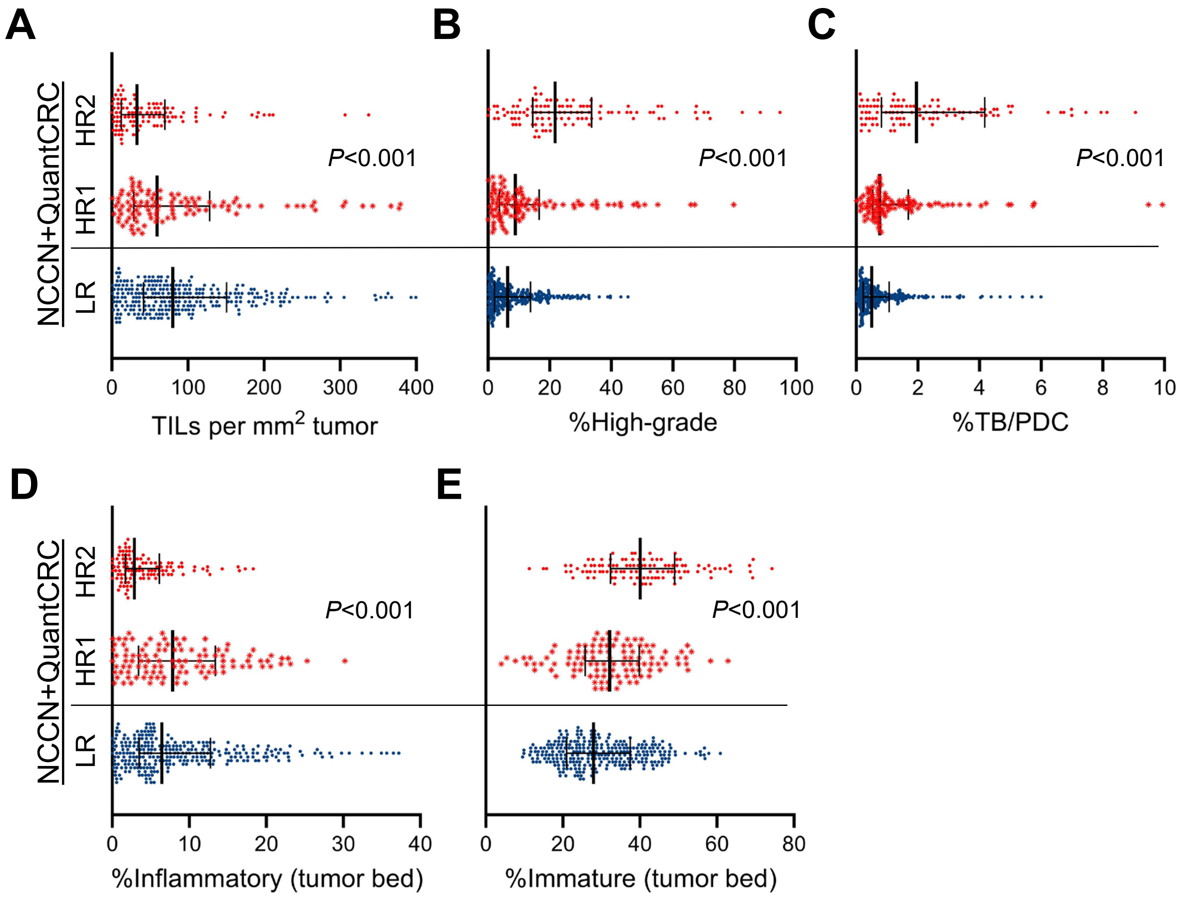

Supplemental Figure 2. QuantCRC features stratified by probability of lymph node metastasis in the NCCN+QuantCRC model. **A-E**. Scatter plots of five QuantCRC features in the NCCN+QuantCRC LR group (predicted probabilities <0.092), NCCN+QuantCRC HR1 (predicted probabilities 0.092-0.218), and NCCN+QuantCRC HR2 (predicted probabilities >0.218). The P-values shown are comparisons between the HR1 and HR2 groups.
