# Supplementary material for: Utility of quantitative pathologic analysis of pT1 colorectal carcinomas to improve prediction of lymph node metastasis
Source: Virchows Arch. 2025 Oct 8;488(6):1319–30. doi: 10.1007/s00428-025-04284-2 (PMC13264598; doi:10.1007/s00428-025-04284-2)
Supplement: Supplementary file 2 — (DOCX 37.7 KB) [file 428_2025_4284_MOESM2_ESM.docx]

**Supplemental Table 1:** Rates of lymph node metastasis according to number and type of NCCN HR features

| **NCCN HR features** | **High risk feature(s)** | **pN+** | ***P*-Value** | |
| --- | --- | --- | --- | --- |
| No HR features (*N*=305) | N/A | 18 (5.9%) | N/A | N/A |
| 1 HR feature (*N*=133) | G3 (*N*=21) | 6 (28.6%) | 0.3 | 0.08 |
|  | Lymphatic invasion (*N*=29) | 7 (24.1%) |  |  |
|  | Bd2/3 (*N*=83) | 13 (15.7%) |  |  |
| 2 HR features (*N*=62) | G3 + Lymphatic invasion (*N*=5) | 0 (0%) | 0.2 |  |
|  | G3 + Bd2/3 (*N*=15) | 4 (26.7%) |  |  |
|  | Lymphatic invasion + Bd2/3 (*N*=42) | 17 (40.5%) |  |  |
| 3 HR features (*N*=12) | High grade + lymphatic invasion + Bd2/3 (N=12) | 4 (33.3%) | N/A |  |

Abbreviations: *HR*, high risk; *NCCN*, National Comprehensive Cancer Network

**Supplemental Table 2:** QuantCRC features according to NCCN and NCCN+QuantCRC risk groups

| **QuantCRC features, median** | **NCCN-LR / NCCN+QuantCRC-LR**  **(*N*=290)** | ***P*-value*** | **NCCN-LR / NCCN+QuantCRC-HR**  **(*N*=15)** | ***P*-value**** | **NCCN HR / NCCN+QuantCRC**  **HR**  **(*N*=207)** |
| --- | --- | --- | --- | --- | --- |
| %Tumor (IQR) | 54.4 (17.8) | 0.1 | 60.2 (16.9) | 0.005 | 50.1 (17.5) |
| %Stroma (IQR) | 40.9 (17.6) | 0.1 | 34.6 (16.5) | 0.002 | 45.9 (17.5) |
| %Mucin within tumor (IQR) | 1.0 (8.6) | 0.5 | 1.1 (21.5) | 0.2 | 0.7 (2.8) |
| %Necrosis (IQR) | 2.6 (4.1) | 0.7 | 1.9 (3.3) | 0.8 | 2.1 (3.1) |
| %TB/PDC (IQR) | 0.5 (0.4) | 0.9 | 0.6 (0.4) | <0.001 | 1.4 (2.2) |
| Tumor:Stroma Ratio (IQR) | 1.3 (1.1) | 0.1 | 1.8 (1.7) | 0.004 | 1.1 (0.8) |
| TILs per mm^2^ (IQR) | 80.3 (109.2) | <0.001 | 22.3 (41.2) | 0.02 | 49.6 (74.2) |
| %High grade (IQR) | 6.3 (11.6) | <0.001 | 43.1 (15.5) | <0.001 | 13.2 (20.4) |
| %SRCC (IQR) | 0.03 (0.1) | 0.02 | 0.2 (1.5) | 0.02 | 0.02 (0.2) |
| %Immature stroma, tumor bed (IQR) | 28.0 (16.6) | 0.7 | 33.0 (21.7) | 0.1 | 37.0 (16.1) |
| %Inflammatory stroma, tumor bed (IQR) | 6.4 (9.2) | <0.001 | 2.1 (4.4) | 0.003 | 5.1 (8.3) |
| %Mature stroma, tumor bed (IQR) | 1.0 (2.5) | 0.9 | 1.0 (2.2) | 0.7 | 1.2 (1.7) |
| %Immature stroma, stromal area (IQR) | 76.1 (24.1) | 0.005 | 92.0 (18.7) | 0.1 | 83.7 (20.0) |
| %Inflammatory stroma, stromal area (IQR) | 17.6 (21.6) | <0.001 | 6.0 (9.9) | 0.04 | 11.1 (17.3) |
| %Mature stroma, stromal area (IQR) | 2.7 (5.4) | 0.7 | 2.3 (9.4) | 0.7 | 2.6 (4.0) |

Abbreviations: *HR*, high risk; *IQR*, interquartile range; *NCCN*, National Comprehensive Cancer Network; LR, low risk; *TB/PDC*, tumor budding/poorly differentiated clusters; *TIL*, tumor infiltrating lymphocyte.

**P*-value comparing NCCN-LR/NCCN+QuantCRC-LR vs. NCCN-LR/NCCN+QuantCRC-HR

***P*-value comparing NCCN-LR/NCCN+QuantCRC-HR vs. NCCN-HR/NCCN+QuantCRC-HR

**Supplemental Table 3:** Differences in QuantCRC features between NCCN+QuantCRC HR1 and HR2 groups

| **QuantCRC features, median** | **NCCN+QuantCRC-HR1**  **(*N*=111)** | **NCCN+QuantCRC-HR2**  **(*N*=111)** | ***P*-value** |
| --- | --- | --- | --- |
| %Tumor (IQR) | 51.1 (16.1) | 49.1 (16.7) | 0.07 |
| %Stroma (IQR) | 45.1 (15.0) | 45.6 (19.2) | 0.2 |
| %Mucin within tumor (IQR) | 0.7 (3.4) | 0.8 (2.9) | 0.6 |
| %Necrosis (IQR) | 1.9 (2.9) | 2.1 (4.0) | 0.4 |
| %TB/PDC (IQR) | 0.8 (1.2) | 2.0 (3.3) | <0.001 |
| Tumor:Stroma Ratio (IQR) | 1.1 (0.8) | 1.1 (0.8) | 0.1 |
| TILs per mm^2^ (IQR) | 59.5 (99.7) | 33.3 (57.2) | <0.001 |
| %High grade (IQR) | 8.8 (12.9) | 21.8 (19.1) | <0.001 |
| %SRCC (IQR) | 0.01 (0.2) | 0.03 (0.3) | 0.2 |
| %Immature stroma, tumor bed (IQR) | 32.1 (14.0) | 40.1 (16.7) | <0.001 |
| %Inflammatory stroma, tumor bed (IQR) | 7.8 (10.0) | 2.9 (4.4) | <0.001 |
| %Mature stroma, tumor bed (IQR) | 1.2 (1.7) | 1.0 (1.8) | 0.8 |
| %Immature stroma, stromal area (IQR) | 79.5 (23.7) | 87.7 (12.8) | <0.001 |
| %Inflammatory stroma, stromal area (IQR) | 16.4 (20.7) | 6.4 (8.5) | <0.001 |
| %Mature stroma, stromal area (IQR) | 2.9 (4.6) | 2.5 (3.8) | 0.5 |

Abbreviations: *HR*, high risk; *IQR*, interquartile range; *NCCN*, National Comprehensive Cancer Network; *LR*, low risk; *TB/PDC*, tumor budding/poorly differentiated clusters; *TIL*, tumor infiltrating lymphocyte.

**Supplemental Table 4:** Pathologic features in the validation cohort of endoscopically resected pT1 CRC followed by surgical resection

| **Pathologic features** | **Endoscopically resected pT1 CRC with pN0 on subsequent surgical resection, *N* (%)** | **Endoscopically resected pT1 CRC with pN+ on subsequent surgical resection, *N* (%)** | ***P*-value** |  |
| --- | --- | --- | --- | --- |
| **Polypectomy margin status**  Negative  Positive  Indeterminate | 9 (42.9)  8 (38.1)  4 (19.0) | 4 (50.0)  4 (50.0)  0 (0) | 0.4 |  |
| **Histologic grade**  G1-2  G3 | 21 (100)  0 (0) | 8 (100)  0 | 1 |  |
| **Lymphatic invasion**  Absent  Present | 15 (71.4)  6 (28.6) | 6 (75.0)  2 (25.0) | 0.6 |  |
| **Tumor budding grade**  Bd1  Bd2/3 | 14 (66.7)  7 (33.3) | 1 (12.5)  7 (87.5) | 0.01 |  |
| **Depth of submucosal invasion, mm (IQR)** | 2.1 (2.8) | 3.0 (2.2) | 0.5 |  |
| **NCCN risk category**  LR  HR | 11 (52.4)  10 (47.6) | 1 (12.5)  7 (87.5) | 0.09 |  |
| **Location**  Proximal  Distal | 5 (23.8)  16 (76.2) | 0 (0)  8 (100) | 0.2 |  |

Abbreviations: Bd, tumor budding; HR, high risk; IQR, interquartile range; LR, low risk; NCCN, National Comprehensive Cancer Network
